# Attenuated Subcomponent Vaccine Design Targeting the SARS-CoV-2 Nucleocapsid Phosphoprotein RNA Binding Domain: *In Silico* Analysis

**DOI:** 10.1155/2020/2837670

**Published:** 2020-09-17

**Authors:** Onyeka S. Chukwudozie, Rebecca C. Chukwuanukwu, Onyekachi O. Iroanya, Daniel M. Eze, Vincent C. Duru, Temiloluwa O. Dele-Alimi, Busuyi D. Kehinde, Taiwo T. Bankole, Perpetua C. Obi, Elizabeth U. Okinedo

**Affiliations:** ^1^Department of Cell Biology and Genetics, University of Lagos, Akoka Lagos State, Nigeria; ^2^Immunology Unit, Medical Laboratory Science Department, Nnamdi Azikiwe University, Nnewi Campus, Nigeria; ^3^Public Health Biotechnology Unit, Institute of Child Health, University College Hospital, University of Ibadan, Nigeria; ^4^Department of Biochemistry, Ladoke Akintola University of Technology, Ogbomosho, Oyo State, Nigeria; ^5^Department of Science Laboratory and Technology (Microbiology Unit), Federal Polytechnic, Oko, Anambra State, Nigeria

## Abstract

The novel coronavirus disease (COVID-19) caused by severe acute respiratory syndrome coronavirus 2 (SARS-CoV-2) has previously never been identified with humans, thereby creating devastation in public health. The need for an effective vaccine to curb this pandemic cannot be overemphasized. In view of this, we designed a subcomponent antigenic peptide vaccine targeting the N-terminal (NT) and C-terminal (CT) RNA binding domains of the nucleocapsid protein that aid in viral replication. Promising antigenic B cell and T cell epitopes were predicted using computational pipelines. The peptides “RIRGGDGKMKDL” and “AFGRRGPEQTQGNFG” were the B cell linear epitopes with good antigenic index and nonallergenic property. Two CD8^+^ and Three CD4^+^ T cell epitopes were also selected considering their safe immunogenic profiling such as allergenicity, antigen level conservancy, antigenicity, peptide toxicity, and putative restrictions to a number of MHC-I and MHC-II alleles. With these selected epitopes, a nonallergenic chimeric peptide vaccine incapable of inducing a type II hypersensitivity reaction was constructed. The molecular interaction between the Toll-like receptor-5 (TLR5) which was triggered by the vaccine was analyzed by molecular docking and scrutinized using dynamics simulation. Finally, *in silico* cloning was performed to ensure the expression and translation efficiency of the vaccine, utilizing the pET-28a vector. This research, therefore, provides a guide for experimental investigation and validation.

## 1. Introduction

Coronavirus disease 2019 (COVID-19) is the disease associated with severe acute respiratory syndrome coronavirus 2 (SARS-CoV-2). Characterizing COVID-19 as a pandemic is an acknowledgment that the coronavirus disease 2019 (COVID-19) outbreak, which started in the Hubei province of China in 2019, has now spread to all continents, affecting most countries around the world with differential impacts and peculiarities [1, 2].

Coronaviruses have the largest known genomes (up to 32 kb) among +RNA viruses, and they encode four structural and sixteen nonstructural proteins [3]. The nonstructural proteins (nsp) consist of all the enzymatic activities that are imperative for viral replication, mostly associated with RNA replication [3, 4]. Structurally, the SARS-CoV-2 genome also encodes an RNA-dependent RNA-polymerase complex consisting of the nsp7, nsp8, and nsp12; the RNA capping machinery which also constitute the nsp10, nsp13, nsp14, and nsp16; and finally additional enzymes such as proteases (the nsp3 PLpro and the nsp5 3CLpro) which cleave viral polyproteins and/or impede innate immunity [5–7].

The four structural proteins together with the viral +RNA genome and the envelope constitute the virion [5, 8]. The matrix (M), small envelope (E), and spike (S) proteins are embedded within the lipid envelope [6, 9]. The fourth structural protein, the nucleocapsid phosphoprotein (N), physically links the envelope to the +RNA genome. It consists of an N-terminal (NTD) and a C-terminal (CTD) domain [10, 11]. Both domains are capable of RNA binding. In addition, the CTD serves as a dimerization domain and binds the matrix protein forming the physical link between the+ RNA genome and the envelope [12]. The SARS N protein has also been shown to modulate the host intracellular machinery and plays regulatory roles during the viral life cycle [5–7]. In light of the genomic similarities between SARS-CoV and SARS-CoV-2, it is reasonable to expect the N protein to function in a similar way [10]. All the SARS-CoV-2 proteins are potential drug and vaccine targets, and a detailed understanding of their functions is therefore of utmost importance. The nucleocapsid phosphoprotein of the severe acute respiratory syndrome coronavirus N (SARS-CoV N) protein packages the viral genome into a helical ribonucleocapsid (RNP) and plays a fundamental role during viral self-assembly [5–7]. It is a protein with multifarious activities. Furthermore, the N protein is frequently used in vaccine development and serological assays [13]. At present, few reports focus on the SARS-CoV-2 N protein, and there is an urgent need for an updated understanding of the SARS-CoV-2 N protein. Majorly, the vaccine therapeutic experiments are highly centered on the spike or entire protein, but we are focusing mainly on the nucleocapsid phosphoprotein which is a protein subset of the virus.

## 2. Materials and Methods

### 2.1. Data Retrieval and Structural and Physiochemical Analysis of SARS-CoV-2 Nucleocapsid Protein

The protein sequence of the SARS-CoV-2 nucleocapsid phosphoprotein was retrieved in a FASTA format from the NCBI repository with the accession numbers: Wuhan, China (GenBank ID: QHD43423.2). The X-ray crystal structure of the nucleocapsid protein was also retrieved from the protein data bank (PDB: 6m3m). The retrieved structure was subjected to a structural alignment to ascertain the level of homology and probable mutations that have occurred over time during viral replication among the coronavirus family. Bootstrap value and other default parameters were used to fabricate the alignment. The physiochemical properties of the protein sequence were assessed and biocomputed via an online tool ProtParam [14] (http://web.expasy.org/protparam/).

### 2.2. Putative B Cell Linear and Discontinuous Epitopes

The nucleocapsid sequence was analyzed with a view to recognize the antigenic regions that were achieved by predicting epitopic peptides. The promising antigenic linear B cell epitopes were predicted using the BepiPred server from the Immune Epitope Database and Analysis Resource (IEDB) database [15]. BepiPred-2.0 is based on a random forest algorithm trained on epitopes annotated from antibody-antigen protein structures [15]. About 12-15 mers (residues) were assumed to bind to the MHC groove. Other criteria such as antigenicity, surface accessibility, flexibility, and hydrophobicity were considered as part of the profiling process of the antigenic B cell linear epitopes. For antigenicity testing, these epitopes were subjected to the VaxiJen 2.0 server at a threshold of 0.6 [16]. The next stage of screening was the prediction of the discontinuous epitopes which are folded in conformation aiding in the antibody recognition of denatured antigens. The ElliPro server (http://tools.iedb.org/ellipro/) was adopted for this purpose, while PyMOL was utilized to examine the positions of forecast epitopes on the 3D structure of SARS-CoV-2 nucleocapsid phosphoprotein [17].

### 2.3. Prediction of Cytotoxic T Lymphocyte (CTL) and Helper T Lymphocyte (HTL) Epitopes

The CTL epitopes were predicted using the IEDB MHC I binding prediction algorithms (http://tools.iedb.org/mhci). This method integrates the prediction of epitopes restricted to a large number of MHC I alleles and proteasomal C-terminal cleavage, using artificial neural network application. For better predictive accuracy, other software such as artificial neural network (ANN), stabilized matrix method (SMM), MHC-binding energy covariance matrix (SMMPMBEC), NetMHCpan, pickpocket, and NetMHCpan were adopted for this purpose. To predict the HTL cell epitopes, the MHC II binding prediction tool (http://tools.iedb.org/mhcii/) found in the IEDB database was adopted. The antigenic properties of the epitopes were studied using the VaxiJen 2.0 server set at a threshold of 0.6. The peptide toxicity predicted from the ToxinPred server (http://crdd.osdd.net/raghava/toxinpred/), allergenicity predicted from AllergenFP 1.0, and digestion predicted from the Protein Digest server were all considered in selecting the final epitopes.

### 2.4. Prediction of the 3D Structures of the Predicted Epitopes and HLA-A 0201 Allele for Molecular Docking

The molecular docking of the antigenic epitopic peptides was conducted with the alleles they were mostly restricted to, which was HLA-A 0201. The protein structure of the allele was retrieved from the protein data bank with the identifier PDB: 4U6Y, while the predicted peptide 3D structures were modeled via the PEP-FOLD server at the RPBS Mobyle portal. The best models provided by the server were chosen for the docking study. The HawkDock Server was employed for the docking process. It combines ATTRACT for global macromolecular docking and HawkRank for scoring.

### 2.5. Homology Modeling of the Conjugated Peptide Vaccine

The three-dimensional model of the conjugated antigenic vaccine was predicted using the I-TASSER server which generates a 3D model of the query sequence by multiple threading alignments and iterative structural assembly simulation [18]. The I-TASSER online server was selected for its availability, composite approach of modeling, and performance in CASP competition. The quality of generated 3D models was checked by *Z* score, and the best model is selected for further consideration. The functional analogs were ranked based on the TM-score, RMSD, sequence identity, and coverage of the structure alignment. The quality of the predicted model was determined by C-score (confidence score) which is ranged as −5 to 2. The obtained 3D model of the conjugated antigenic vaccine and the human Toll-like receptor-5 PDB structures were aligned employing the TM-align [19], a quick and accurate structural alignment tool for two protein structures of unknown equivalence. An optimal superposition of the two structures built on the detected alignment, as well as the TM-score value which scales the structural similarity, will be returned. TM-score has the value in (0,1), where 1 indicates a perfect match between two structures.

### 2.6. Validation of Predicted Conjugated Peptide Vaccine 3D Model

The confirmation of the selected 3D model predicted by I-TASSER was further validated by the Ramachandran plot. RAMPAGE and MolProbity [20] online servers were employed for the estimation of selected 3D model quality. It can begin from either the C-alpha trace, main-chain model, or full-atomic model. The Ramachandran plot obtained from RAMPAGE describes a good quality model that has over 70% residues in the most favored region. The plot analysis was able to show the allowed and disallowed dihedral angles psi (*ψ*) and phi (*ϕ*) of an amino acid which is calculated based on the van der Waal radius of the side chain. The corresponding percentage value of both the allowed and disallowed regions of the separate plots of glycine and proline residues of the modeled structure was generated. Qualitative evaluation of 3D models was employed by ProSA [21]. ProSA specifically faces the needs confronted in the authentication of protein structures acquired from X-ray analysis, NMR spectroscopy, and hypothetical estimations.

### 2.7. Protein-Protein Docking of the Peptide Vaccine and the Human Toll-Like Receptor-5 (TLR5)

In this study, molecular docking analysis between the vaccine and the human Toll-like receptor-5 was performed using the ClusPro 2.2 protein-protein interaction online server [22]. The shape complementarity and minimal binding energy of the Toll-like receptor-5 with predicted conjugated antigenic vaccine model obtained from I-TASSER is determined by the cluster scores for the lowest binding energy prediction, calculated using the following formula [23]:

(1) $$\begin{array}{c} -E=0.40E\;\left[\text{rep}\right]+-0.40E\;\left[\text{att}\right]+600E\;\left[\text{elec}\right]+1.00E\;\left[\text{DARS}\right]. \end{array}$$

Here, repulsive, attractive, and electrostatic as well as interactions extracted from the decoys as the reference state are considered for structure-based pairwise potential calculation in docking [22]. The best PDB conformation was subjected to the Prodigy server to ascertain the binding free energy of the protein complex.

### 2.8. Molecular Dynamics Simulations

The interacting complex between the vaccine and the Toll-like receptor (PDB: 3J0A) was thoroughly accessed based on the existing coordinates between the docked protein complex. Parameters considered were the deformability, B factor, and eigenvalues associated with the normal mode which represents the motion stiffness. The lower the eigenvalue, the easier the deformation. The covariance matrix was also considered for the simulation. It indicates the coupling between the pairs of residues. The correlation matrix is computed using the C*α* Cartesian coordinates [24].

### 2.9. Codon Optimization and *In Silico* Cloning

A codon optimization was conducted to ascertain the maximum expression of the vaccine in the host. This was done with the aim of boosting the vaccine translational rate in *E. coli* K12. Restriction enzyme cleavage sites, prokaryote ribosomal binding site, and finally rho-independent transcription termination were all avoided during the option selection. Codon adaptation index (CAI) value and GC content of the adapted sequence was obtained and compared with the ideal range. The obtained refined nucleotide was cloned into the pET28a D315A vector.

## 3. Results

### 3.1. Structural Alignment Studies of the SARS-CoV-2 NP

**Figure 1:**
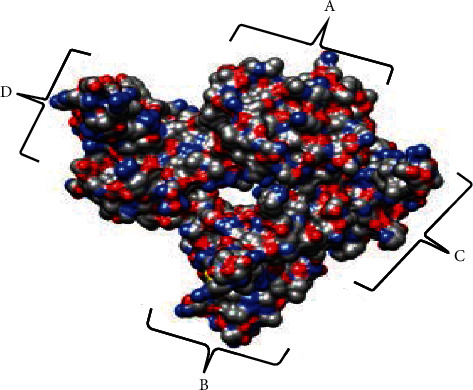
Crystal structure of SARS-CoV-2 nucleocapsid protein N-terminal RNA binding domain at a resolution of 2.70 Å (PDB: 6m3m). The chain components are identified.

**Figure 2:**
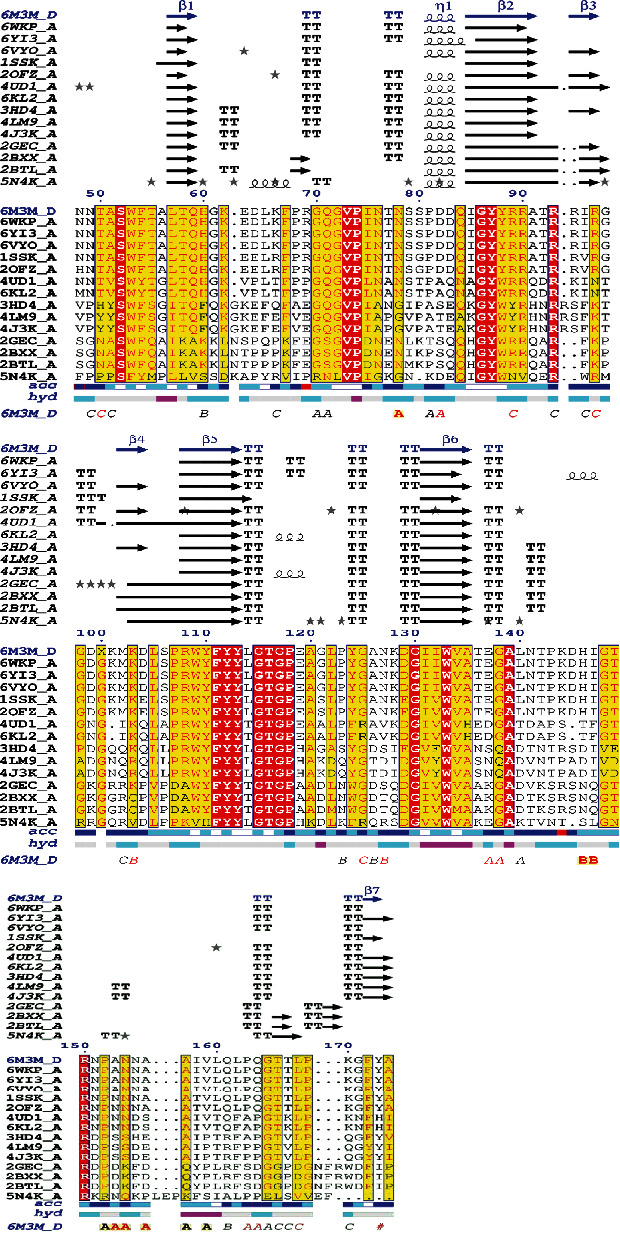
Structural alignment of the SARS-CoV-2 RNA binding domain of nucleocapsid phosphoprotein. Helices are represented in squiggles, while beta strands with arrows and turns with TT letters. Solvent accessibility is rendered by a first bar below the sequence (blue is accessible, cyan is intermediate, and white is buried) and hydropathy by a second bar below (pink is hydrophobic, white is neutral, and cyan is hydrophilic). Bottom letters and symbols depict crystallographicity. Alignments in red represent conserved regions, yellow highlights the regions that tend towards monomorphism, while white is regions that are highly mutated. The dotted segments are the sequence deletions.

The protein structure consists of 4 side chains as shown, which suggests a plausible model for RNA binding (Figure 1). A structural alignment was performed to ascertain the level of conservancy across the coronavirus NP, while the SARS-CoV-2 (PDB: 6m3m) was maintained as the reference protein. Sequence similarity search and multiple sequence alignment (MSA) were adopted for this purpose. The MSA of the 14 homologous proteins to 6m3m was generated with a BLAST search against the PDBAA database. Across the sequence alignment, the various mutations including deletions supersede the conserved regions of the amino residues (Figure 2).

In summary, the alignment construct was less conserved, signifying the rapid rate of mutations that has acted on the protein during viral replications. The protein identification of the PDB IDs is summarized (supplementary file: Table 1). Also, results of the solvent accessibility and hydropathy of the N NTD of coronaviruses show that most of the regions on the sequence have high solvent accessibility and are hydrophilic, properties which make the N NTD a likely antigenic target.

### 3.2. B Cell Linear and Discontinuous Epitopes

**Figure 3:**
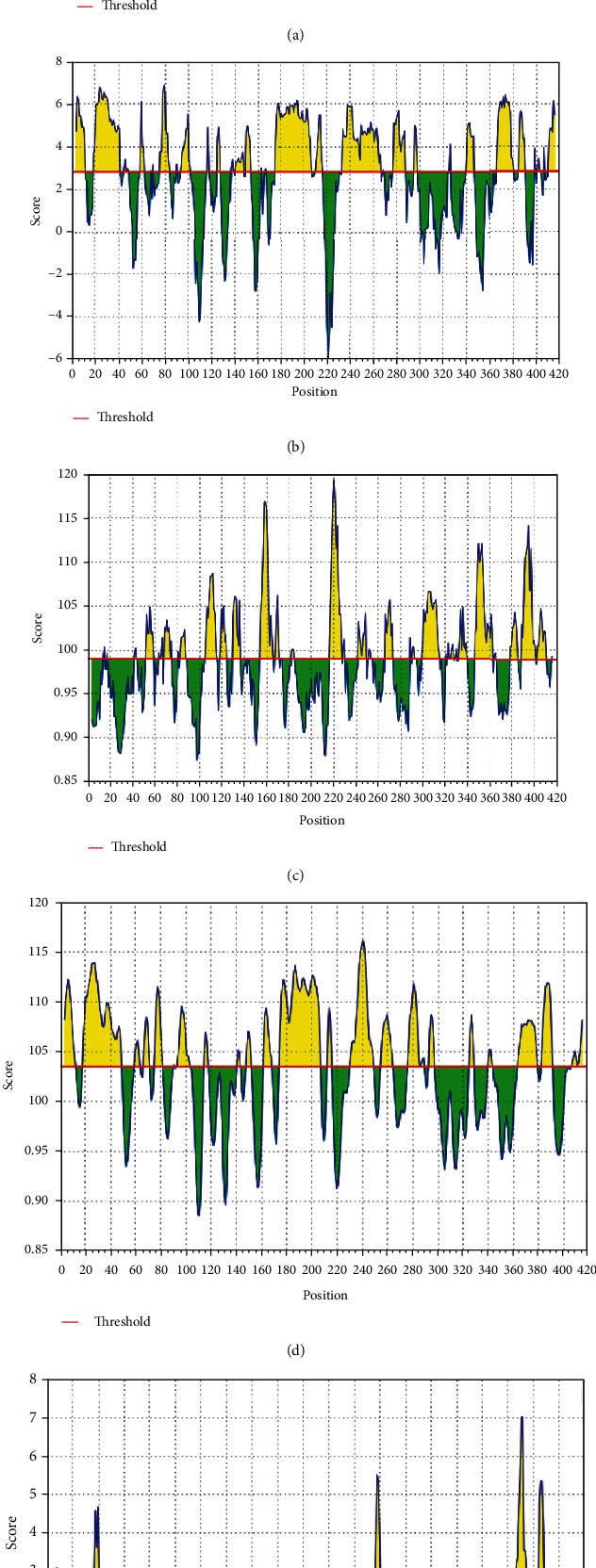
(a–f) The properties of the B cell linear epitope predictions: (a) the linear epitopes, (b) hydrophobicity, (c) antigenicity, (d) flexibility, and (e) surface accessibility. Green regions under the threshold color denote unfavorableness related to the properties of interest. Yellow colors are above the threshold sharing higher scores. Horizontal red lines represent the threshold.

**Figure 4:**
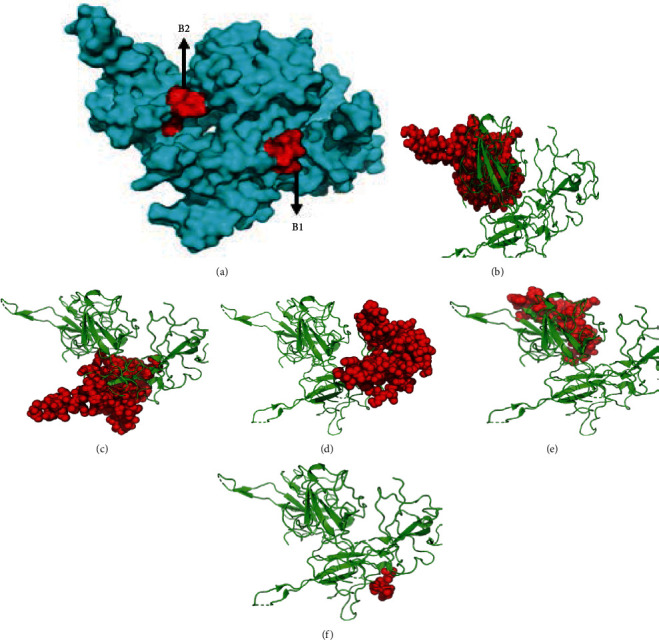
(a) Mapped out antigenic B cell linear epitopes (red) of the SARS-CoV-2 nucleocapsid protein. (b–f) The antibody recognition of SARS-CoV-2 denatured antigens (red spheres) in (b) D monomer, (c) B monomer, (d) C monomer, (e) A monomer, and (f) B monomer.

Utilizing the Kolaskar and Tongaonkar antigenicity scale, Emini surface accessibility, and Chou and Fasman beta turn predictions, regions with viable antigenic properties were predicted. This scale was able to show the favorable regions across the protein that are potentially antigenic (Figures 3(a)–3(e)). A total of four antigenic B cell epitopes was predicted. These epitopes had a safe physiochemical property such as the absence of peptide toxicity and lesser allergenicity, making it safe for vaccine production. Three of the selected epitopes had 100% across the antigen. Based on the conservancy across the antigen and the allergenicity, only RIRGGDGKMKDL and AFGRRGPEQTQGNFG were selected as the final promising antigenic B cell linear epitopes (Table 1). These epitopes were mapped out from the protein structure (Figure 4(a)). Based on the SARS-CoV-2 nucleocapsid protein (PDB: 6m3m), the discontinuous epitopes were predicted considering their propensity scores. The identified denatured antigens by the neutralizing antibody are highlighted (Figures 4(b)–4(f)). The residues are juxtaposed enabling the antibody to recognize the 3D dimensional structure. The chain D component of the protein had the highest denatured antigens with a total of 81 residues, followed by chains B, C, and A, in that hierarchical order as the number of residues and their respective rank scores are ranked (Table 2). A higher propensity score of 0.745 means that only 25% of the D-chain residues are nonepitope residues predicted as part of the epitope. Also, the result of the linear B cell epitope prediction correlated with the discontinuous epitope as the dodecapeptide epitope predicted in the B cell linear epitope was also found to be in the D-chain component of the protein, further indicating the antigenicity of the D-chain monomer of the N NTD.

### 3.3. The CTL and HTL Epitopes

Two nonallergenic and nontoxic cytotoxic epitopes, with a viable antigenic property, were selected. The epitopes are GMSRIGMEV and LTYTGAIKL (Table 3). For the helper T cell epitopes, three were selected based on their conservancy score of 100%, nonallergenic attributes, and their respective antigenic properties (Table 4). Few of the peptides, regardless of their antigenic nature, had an allergenic property capable of inducing a harmful autoimmune response. The promiscuity of the chosen peptides was also evaluated by considering the number of MHC-I and MHC-II alleles they are putatively restricted to.

### 3.4. Molecular Docking of HLA-Epitope Interaction with the MHC-I Molecule

**Figure 5:**
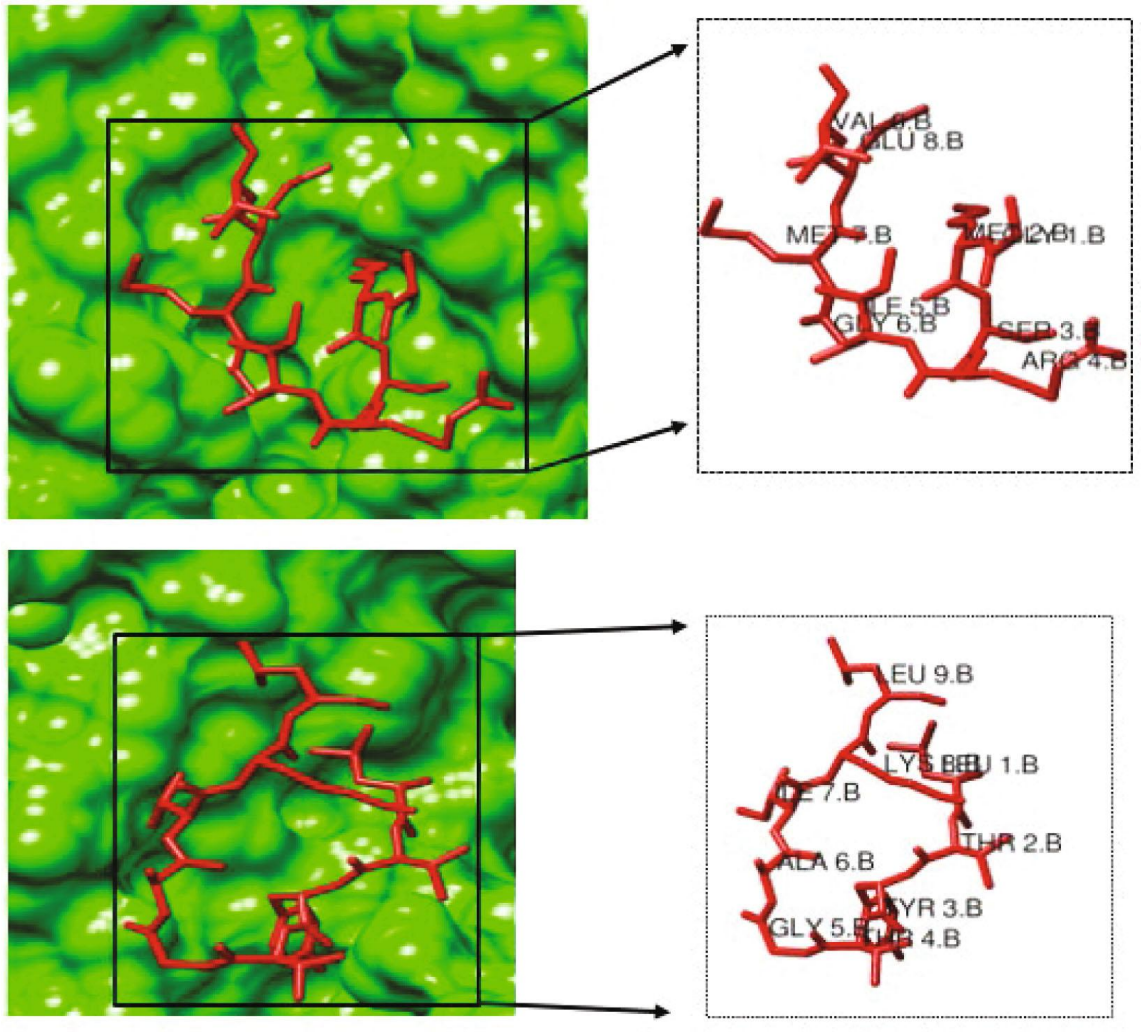
Molecular docking of the peptides GMSRIGMEV and LTYTGAIKL and the HLA A0201 molecule. GMSRIGMEV had binding free energy of -8.3 kcal/mol and -10.0 kcal/mol for LTYTGAIKL.

Both peptides “GMSRIGMEV and LTYTGAIKL” bind, respectively, to their restricted HLA molecules. Both epitopic peptides were highly restricted to several MHC-I molecules. In the case of HLA-A 0201, the binding free energy of GMSRIGMEV with the MHC-I antigen-binding groove was -8.3 kcal/mol, while the peptide LTYTGAIKL had binding energy of -10 kcal/mol (Figure 5).

### 3.5. Eminent Profiling of the Chimeric Vaccine Construct

The final conjugated vaccine consists of two B cell linear epitopes, two CD4+, three CD8+ epitopes, and a polyhistidine tag, which sums up the total of 78 amino residues. Given the high antigenicity index of 0.75 of the predicted vaccine, viable enough in eliciting both humoral and cellular immune responses, and their nontoxicity and allergenicity, an immunoadjuvant to boost the antigenicity of the vaccine construct was excluded. The addition of polyhistidine residues at the C-terminal of the vaccine helps to convey increased purity to the recombinant protein and may contain specified epitopes that can be recognized by an antibody fragment, thereby increasing the efficacy and effectiveness of the peptide vaccine.

### 3.6. Physiochemical and Solubility Properties of the Vaccine

**Figure 6:**
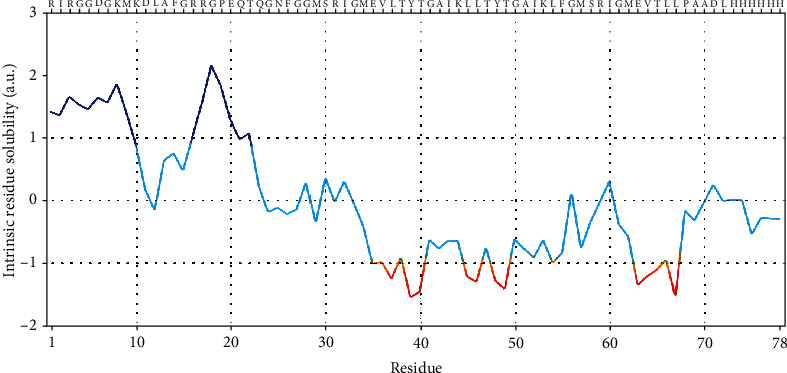
Solubility index profile of the peptide vaccine. Residues less than -1 depict the hydrophobic core of the vaccine peptide.

The molecular weight of the vaccine was 8558.93 Da, and the biocomputed theoretical pI was 9.98, with an estimated half-life of 30 hours. The instability index was 30.09, signifying that the protein is stable (>40 signifies instability). The aliphatic index is computed to be 78.85, with a GRAVY score of -0.281, signifying its hydrophilic nature. The atomic composition of the vaccine is 376 carbon, 603 hydrogen, 115 nitrogen, 104 oxygen, and 5 sulfur, thereby giving rise to the chemical formula C_376_H_603_N_115_O_104_S_5,_ with a total of 1203 atoms. The extinction coefficient at a wavelength of 280 nm was 2980 M^−1^ cm^−1^. The intrinsic vaccine solubility at a neutral pH 7 revealed the hydrophilic and hydrophobic core of the vaccine construct (Figure 6).

### 3.7. Three-Dimensional Structure Prediction of the Vaccine

**Figure 7:**
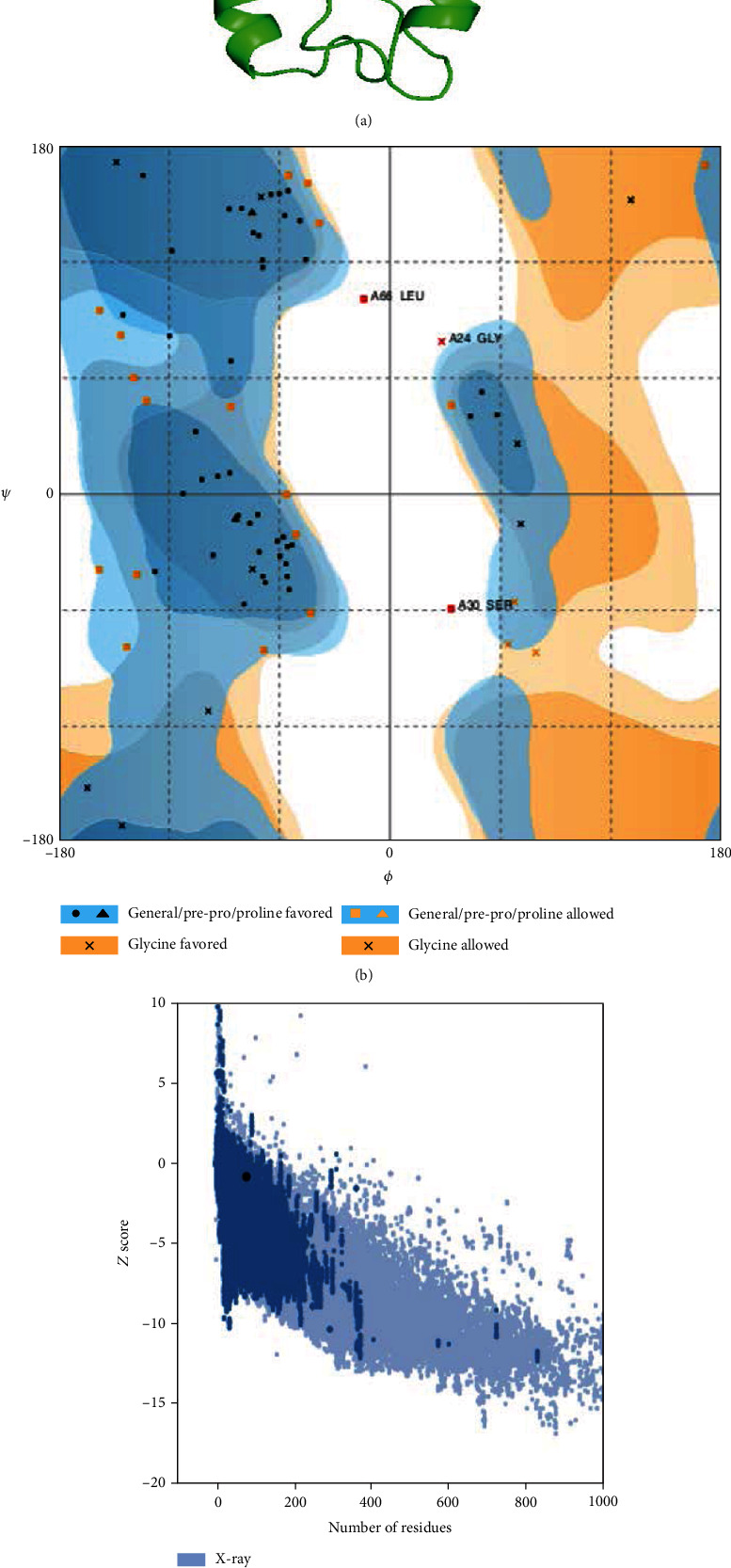
(a) Three-dimensional structure of vaccine predicted by I-TASSER. Three-dimensional structure prediction validation of top score model of I-TASSER by (b) RAMPAGE assessment of the Ramachandran plot of the selected model. Number of residues in favored region: 53 (69.7%), number of residues in allowed region: 20 (26.3%), number of residues in outlier region: 3 (3.9%). (c) ProSA protein structure analysis results. *Z* score = −0.79. Overall quality of the ultimate model is acceptable.

The predicted model of the vaccine and its three-dimensional coordinate file was successfully obtained from I-TASSER (Figure 7(a)). The results obtained from the server includes the predicted secondary structure with a confidence score ranging from 0 to 9, predicted solvent accessibility, functional analogs protein, and binding site residues. The best model was selected with a C-score of -1.39, TM-score of 0.54 ± 0.15, and RMSD at 6.3 ± 3.8 Å.

### 3.8. Structural Validation of the Predicted Model

The Ramachandran plots of the predicted model were obtained to verify the stereochemical parameters of the protein structure. The Ramachandran plot showed 69.7% residues in most favored regions and 26.3% residues in additional allowed regions, i.e., the total of 96% residues in allowed regions which indicates a good quality model (Figure 7(b)); this was also attested to by the MolProbity Ramachandran plot which also showed 96.6% residues in allowed regions which also confirmed the quality of the predicted model (Figure 7(c)).

### 3.9. Molecular Interaction between the Peptide Vaccine and the Toll-Like Receptor-5

**Figure 8:**
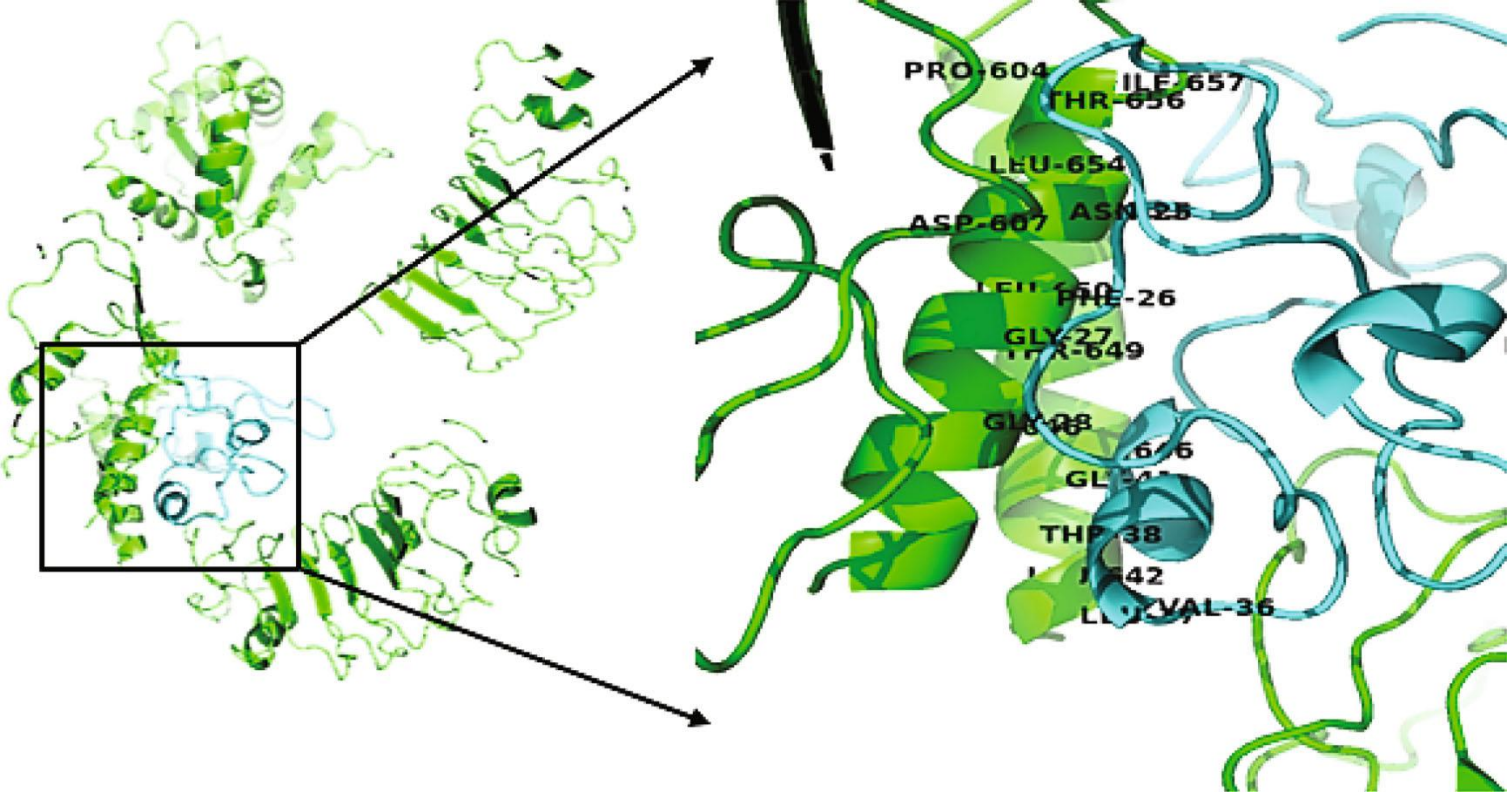
The interaction of the proposed vaccine construct with TLR5. The vaccine and TLR5 are shown in red and blue color, respectively. The blue color signifies the vaccine, while the green color also signifies the chain of the receptor.

A preliminary docking preparation was conducted by aligning the vaccine construct with the Toll-like receptor to ascertain if both complexes are likely to interact. The TM alignment score obtained was 0.35240, which shows the likelihood of both proteins interacting with stable conformation. The docking structure of the human Toll-like receptor-5 binding with peptide vaccine fragment was finally obtained. A conformational change occurs in the Toll-like receptor-5 protein after binding with the antigenic peptide (Figure 8), with a binding energy of -8.6 kcal/mol. The interface residue contacts characterizing both protein interactions include the following: charged-charged was 7, charged-polar was 6, charged-apolar was 11, polar-polar was set at 0, polar-apolar was 7, and finally, apolar-apolar was 20. The noninteracting charged surface was 20.26%, while the noninteracting apolar surface was 42.41%. The interacting amino residues of the vaccine comprised of ASN-25, PHE-26, GLY-27, GLY-28, VAL-35, LEU-36, and THR-37 were able to form a hydrogen bond with PRO-604.A, ASP-607.A, CYS-646.A, LEU-650.A, PHE-653.A, LEU-654.A, LEU-642.B, CYS-646.B, and THR-649.B of the receptor.

### 3.10. Molecular Dynamics Simulations

**Figure 9:**
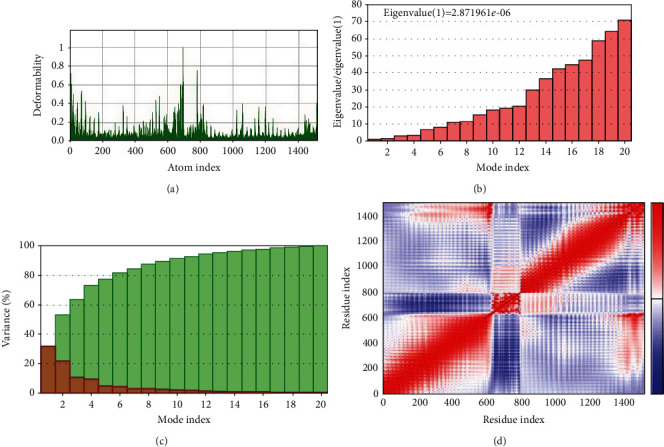
(a–d) Molecular dynamics simulation of the vaccine-TLR5 complex, showing (a) eigenvalue, (b) deformability, and (c) B factor, and (d) covariance matrix indicates coupling between pairs of residues, i.e., whether they experience correlated (red), uncorrelated (white), or anticorrelated (blue) motions.

The interaction between the peptide vaccine and the Toll-like receptor was scrutinized to check for their protein stability and deformation. This analysis relies on the associated coordinates of the docked protein complex (Figures 9(a)–9(d)). The eigenvalue found for the complex was 2.871961*e*-06. The low eigenvalue for the complex signifies easier deformation of the complex, indicating that the docking analysis between the vaccine and the TLR5 will activate immune cascades for destroying the viral antigens.

### 3.11. Codon Optimization and *In Silico* Cloning

**Figure 10:**
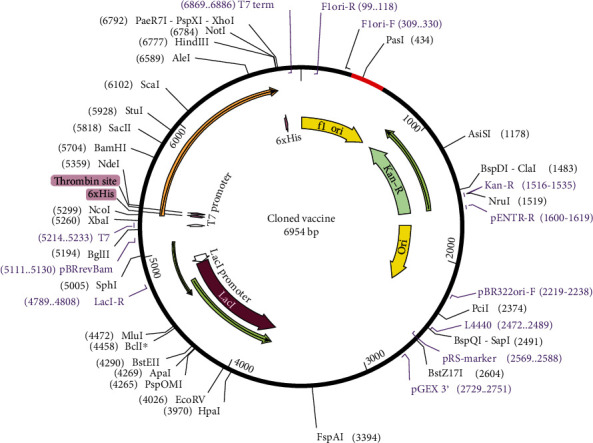
*In silico* cloning of the final vaccine construct into an expression vector where the red part indicates the coding gene for the vaccine enzymatically restricted by PasI.

The length of the optimized vaccine codon sequence was 234 nucleotides. The GC content of the improved cDNA sequence was 54.27%, which still falls within the recommended range of 30-70%, for effective translational efficiency. The codon adaptive index was calculated as 1.0, falling within the range of 0.8-1.0, signifying the effective expression of the vaccine construct in the *E. coli*. EagI-NotI and SAlI sites were subsequently cloned into the pET28a D315A vector. The estimated length of the clone was 6.954 kbp (Figure 10).

## 4. Discussion

The menace of the coronavirus pandemic on global health has made imperative the development of safe, stable, and effective vaccines against it. Currently, most vaccine designs against the SARS-COV-2 virus are targeted against the viral antigenic proteins (spike and nucleocapsid) either as whole inactivated or attenuated viruses because of its ability to elicit neutralizing antibodies to block virus-receptor interaction and neutralize the viral infection of cells [25, 26]. Unlike the spike protein that induces high neutralizing antibodies which are incapable of inducing long-lasting protection against the virus, the nucleocapsid protein does not elicit neutralizing antibodies but may induce specific antibody and cellular immune responses [7, 27]. This was the primary focus of this research in predicting antigenic peptides as potential vaccine candidates that would provide a long-lasting cellular immunity against the virus. Also, the N protein is more conserved and induces long-lived memory T cells in humans, features that make it a potent vaccine candidate [27–30]. In this study, we explored the potentials of the nucleocapsid phosphoprotein RNA binding domain as a subcomponent vaccine candidate which could induce protective immunity against the SARS-COV-2 virus.

Observations from previous studies have shown the conservation level and immunogenic properties of the SARS-CoV-2 N protein. According to their reports, the N protein as a vaccine target has specific advantages over the spike, because of its conservancy, resulting in less mutation of the epitope sequence [31]. Our structural alignments corroborate that the N protein is moderately conserved (Figure 2). This could be attributed to the mutation of the N protein sequence in the coronavirus family. Dawood reported a moderate mutation of the N protein of coronaviruses [31]. This is indicative that the SARS-COV-2 virus may have originated from mutations within the family as has been previously suggested [32].

The humoral immune response plays a clear role in vaccine-mediated defense against infections given their role in maintaining memory cells, prolonged survival, and sentinel against reinfection [33]. Subcomponent vaccines that are capable of focusing the humoral immune response on specific antigenic epitopes can be predicted to be crucial and most beneficial in inducing specific antibodies and long-lived memory immune cells. Here, we predicted 2 viable antigenic regions using diverse investigation tools and processes for the calculation of the B cell linear epitopes. These 2 regions (one a dodecapeptide and the other a pentadecapeptide) were selected based on their conservancy, high antigenicity, nontoxicity, and nonallergenic properties. Due to findings from the diverse investigation tools employed, we report that our identified epitopes can elicit humoral immunity. This is in consonance with previous studies [34, 35] who reported the ability of the SARS-COV N protein to elicit N-specific humoral immune response *in vitro*. Also, the discontinuous B cell epitope prediction, which is amino acid residues that were brought into close proximity within the folded protein structure [36], revealed that the chain D component of the protein had the highest propensity score. This is important because antibody binding is not just determined by the linear peptide segment but is also influenced by adjacent surface regions [37, 38].

The outcome of disease infection in humans is usually a factor of the strength of the immune response mounted against the infectious agent, and this is usually orchestrated by MHC molecules of the cellular immune system [32]. While B cells recognize epitopes on the surface of the infectious agent, T cells recognize epitopes on MHCs. Two subpopulations of T cells (cytotoxic T lymphocytes and helper T lymphocytes) are involved in epitope recognition, and while CD8+ CTLs recognize antigens presented on MHC class I, CD4+ HTLs recognize antigens on MHC class II. Also, CD4+ HTLs play a vital role in coordinating both humoral and cell-mediated immune responses [39–41]. The two CTLs (C_2_ and C_3_) were selected due to their high antigenicity among the four identified epitopes. The three HTL epitopes (H_2_, H_4_, and H_6_) were selected based on their nonallergenicity and high antigenicity. Even though other epitopes had higher antigenicity scores, they were however found to be allergenic, able to elicit a harmful autoimmune response. Therefore, they were not selected.

Interestingly, a recent study predicted some T cell epitopes which can be recognized by MHC class II CD4+ HTL alleles of the Asian and Asia-Pacific region populations [32]. While they considered only the MHC class II epitopes, we considered both MHC class I and II epitopes in our study. Also, no HTL epitope was identified in their nucleocapsid protein; however, we report that two CTL and three HTL epitopes with high antigenicity and absence of allergenicity were identified on the nucleocapsid protein N terminal RNA binding domain of SARS-COV-2. Also similar to our study, Wang et al. predicted 4 strong antigenic sites and synthesized 2 strong immunogenic peptides on the N protein of SARS-CoV, of which one of our predicted peptides falls within the synthesized peptides showing medium-strong immunogenicity [42]. However, our study also revealed epitopes which have not been reported earlier. This indicates that the N protein is one of the major antigens of the coronaviruses with diverse potent epitopes for vaccine development.

We examined the interaction of the designed vaccine with TLR5 using molecular docking analysis. The binding interfaces between the TLR5 and the peptide vaccine consisted of hydrogen bonding and hydrophobic interactions. Also, the relative binding free energies of the vaccine-TLR5 complex suggests that the linking of the peptide vaccine construct to the receptor elicits conformational changes that favor stimulation of the TLR5 immune molecules and indicate a favorable protein-protein interaction of our vaccine construct with the innate immune receptor. Similarly, the result of the molecular simulation analysis to examine for stability and deformation in the interaction between the vaccine and the TLR5 complex found a low eigenvalue for the complex indicating easier deformation of the complex. This implies that the docking analysis between the vaccine and the TLR5 will activate immune cascades for destroying the viral antigens. We therefore report that the *in silico* epitope-based vaccine construct targeting the SARS-COV-2 N protein N-terminal RNA binding domain shows prospects as a potent, safe, and effective candidate with high antigenic properties and a balanced immune response operating through both innate and adaptive pathways.

For *in vitro* and *in vivo* studies to fully validate this research, selected antigenic and nonallergenic peptides that were predicted from this *in silico* simulation can be synthesized and tested to corroborate our preliminary results.

## 5. Conclusion

The lack of an effective therapeutic candidate against the novel coronavirus has created a huge task for biomedical researchers to seek research approaches for overcoming the pandemic. This study was designed in furtherance of steps towards vaccine development. We used the primary amino acid sequence of the SARS-COV-2 to design a subcomponent peptide vaccine construct. The vaccine construct has both adaptive (B and T cell) epitopes and a favorable interaction with the pathogen recognition receptors (PRRs) (via TLR5) of the innate immune system. Each of the predicted epitopes has antigenic properties in the absence of allergenic properties. Generally, this study applied a series of immunoinformatic tools to predict a safe, stable, and effective peptide vaccine that may fight against the SARS-COV-2 viral infection. However, we propose experimental validations to prove this computational work.

## Figures and Tables

**Table 1:** B cell linear epitopes of SARS-CoV-2 NP and their toxicity properties.

No.	Start	End	Peptide	Length	Antigenicity	Conservancy	Toxicity	Allergenicity
1	93	104	RIRGGDGKMKDL^∗^	12	0.8771	100	Nontoxic	Nonallergen
2	273	278	AFGRRGPEQTQGNFG^∗^	15	1.1728	100	Nontoxic	Nonallergen
3	338	347	LDDKDPNFK	10	2.1298	100	Nontoxic	Allergen
4	91	110	ATRRIRGDGKMKDLSPRWY	19	0.7147	0	Nontoxic	Nonallergen

^∗^Selected B cell linear epitopes. Selected epitopes are tagged as B1 and B2.

**Table 2:** The B cell discontinuous epitopes of the SARS-CoV-2 nucleocapsid phosphoprotein.

No.	Residues	Number of residues	Score	3d structure
1	D:A56, D:L57, D:T58, D:Q59, D:H60, D:G61, D:K62, D:E63, D:D64, D:L65, D:K66, D:F67, D:P68, D:R69, D:G70, D:Q71, D:G72, D:V73, D:P74, D:Q84, D:Y88, D:R90, D:A91, D:T92, D:R93, D:R94, D:I95, D:R96, D:G97, D:G98, D:D99, D:K101, D:M102, D:K103, D:D104, D:L105, D:S106, D:P107, D:R108, D:W109, D:G117, D:P118, D:E119, D:A120, D:G121, D:L122, D:P123, D:Y124, D:G125, D:A126, D:N127, D:K128, D:D129, D:G130, D:I131, D:I132, D:W133, D:V134, D:A135, D:T136, D:E137, D:G138, D:A139, D:L140, D:N141, D:T142, D:P143, D:Q161, D:L162, D:P163, D:Q164, D:G165, D:T166, D:T167, D:L168, D:P169, D:K170, D:G171, D:F172, D:Y173, D:A174	81	0.745	Figure 4(b)
2	B:N48, B:N49, B:T50, B:A51, B:S52, B:W53, B:F54, B:T55, B:A56, B:T58, B:Q59, B:H60, B:G61, B:P74, B:A91, B:T92, B:R93, B:R94, B:I95, B:R96, B:G97, B:D99, B:G100, B:K101, B:M102, B:K103, B:D104, B:L105, B:S106, B:P107, B:R108, B:Y110, B:L114, B:G115, B:T116, B:G117, B:P118, B:E119, B:A120, B:T142, B:P143, B:K144, B:D145, B:H146, B:I147, B:G148, B:T149, B:R150, B:N151, B:P152, B:A153, B:N154, B:N155, B:A156, B:A157, B:I158, B:V159, B:L160, B:G171	59	0.692	Figure 4(c)
3	C:P47, C:N48, C:N49, C:T50, C:A51, C:W53, C:Q59, C:H60, C:G61, C:K62, C:E63, C:D64, C:L65, C:K66, C:F67, C:P68, C:R69, C:G70, C:Q71, C:G72, C:V73, C:S79, C:S80, C:P81, C:D82, C:D83, C:Q84, C:I85, C:G86, C:Y87, C:R89, C:R90, C:A91, C:T92, C:R93, C:R94, C:I95, C:R96, C:G98, C:D99, C:G100, C:K101, C:M102, C:K103, C:D104, C:L105, C:S106, C:P107, C:Y113, C:L114, C:G115, C:T116, C:G117, C:P118, C:E119, C:A120, C:G121, C:L122, C:P123, C:Y124, C:G125, C:A126, C:N127, C:K128, C:D129, C:G130, C:I131, C:I132, C:W133, C:V134, C:A135, C:T136, C:E137, C:G138, C:A139, C:L140, C:N141, C:T142, C:P143, C:K144, C:D145, C:H146, C:I147, C:G148, C:T149, C:R150, C:N151, C:P152, C:A153, C:N154, C:N155, C:T166, C:G171	93	0.683	Figure 4(d)
4	A:H60, A:G61, A:K62, A:E63, A:D64, A:L65, A:K66, A:F67, A:P68, A:R69, A:G70, A:Q71, A:G72, A:V73, A:S79, A:S80, A:P81, A:D82, A:Q84, A:A135, A:T136, A:E137, A:G138, A:A139, A:L140, A:N141, A:T142, A:Q161, A:L162, A:P163, A:Q164, A:G165, A:T166, A:T167, A:L168, A:P169, A:K170, A:G171, A:Y173	39	0.658	Figure 4(e)
5	B:I75, B:N76, B:T77, B:N78, B:S79, B:S80	6	0.617	Figure 4(f)

**Table 3:** MHC-I epitopes of SARS-CoV-2 NP.

S/N	Start	End	Peptide	Length	Alleles	Antigenicity	Toxicity	Allergenicity
1	222	230	LLLDRLNQL	9	HLA-A∗02:01, HLA-C∗03:02, HLA-C∗01:02, HLA-A∗30:01, HLA-B∗07:02, HLA-A∗01:01, HLA-B∗35:01, HLA-A∗03:01	0.1566	Nontoxic	Nonallergen
2	316	324	GMSRIGMEV^∗^	9	HLA-A∗02:01, HLA-C∗01:02, HLA-C∗03:02, HLA-A∗30:01, HLA-A∗01:01, HLA-A∗03:01, HLA-B∗07:02, HLA-B∗35:01	0.6287	Nontoxic	Nonallergen
3	331	339	LTYTGAIKL^∗^	9	HLA-A∗02:01, HLA-C∗01:02, HLA-C∗03:02, HLA-A∗30:01, HLA-A∗01:01, HLA-A∗03:01, HLA-B∗07:02, HLA-B∗35:01	0.6524	Nontoxic	Nonallergen
4	406	414	QLQQSMSSA	9	HLA-A∗03:01, HLA-B∗07:02, HLA-B∗35:01	0.3180	Nontoxic	Nonallergen

^∗^Selected CTL epitope. Selected epitopes are tagged as C2 and C3.

**Table 4:** MHC-II epitopes of SARS-CoV-2 NP.

S/N	Start	End	Peptide	Length	Core sequence	Alleles	Antigenicity	Conservancy	Toxicity	Allergenicity
1	107	121	YYLGTGPEA	15	RWYFYYLGTGPEAGL	—	0.7969	100	Nontoxic	Allergen
2	325	339	LTYTGAIKL	15	TPSGTWLTYTGAIKL^∗^	HLA-DRB1∗07:01, HLA-DRB5∗01:01, HLA-DRB1∗15:01, HLA-DRB3∗02:02	0.6524	100	Nontoxic	Nonallergen
3	106	120	FYYLGTGPE	15	PRWYFYYLGTGPEAG	—	1.1904	100	Nontoxic	Allergen
4	314	328	FGMSRIGME	15	FFGMSRIGMEVTPSG^∗^	HLA-DRB4∗01:01, HLA-DRB3∗02:02, HLA-DRB1∗03:01, HLA-DRB1∗15:01	0.9467	100	Nontoxic	Nonallergen
5	222	236	LNQLESKMS	15	LLLDRLNQLESKMSG	—	0.6213	100	Nontoxic	Allergen
6	387	401	VTLLPAADL	15	KKQQTVTLLPAADLD^∗^	HLA-DRB4∗01:01, HLA-DRB5∗01:01, HLA-DRB1∗07:01, HLA-DRB1∗15:01	0.6417	100	Nontoxic	Nonallergen

^∗^Selected HTL epitopes. Selected epitopes are tagged as H2, H4, and H6.

## Data Availability

Data are available upon request and may be obtained by contacting the corresponding author.

## Supplementary Materials

Supplementary Materials Supplementary Table 1: summarized nucleocapsid protein structures that were automatically obtained via the PDBAA database with BLAST with the SARS-COV-2 nucleocapsid protein. The proteins are all within the coronavirus family.
