# Supplementary material for: Attenuated Subcomponent Vaccine Design Targeting the SARS-CoV-2 Nucleocapsid Phosphoprotein RNA Binding Domain: In Silico Analysis
Source: J Immunol Res. 2020 Sep 17;2020:2837670. doi: 10.1155/2020/2837670 (PMC7501546; doi:10.1155/2020/2837670)
Supplement: Supplementary Materials — Supplementary Table 1: summarized nucleocapsid protein structures that were automatically obtained via the PDBAA database with BLAST with the SARS-COV-2 nucleocapsid protein. The proteins are all within the coronavirus family. [file 2837670.f1.docx]

**Supplementary file**

**Table 1**

| **S/N** | **PDB ID** | **Protein name** | **Year Released** |
| --- | --- | --- | --- |
| 1. | 6M3M | [Crystal structure of SARS-CoV-2 nucleocapsid protein N-terminal RNA binding domain](https://www.rcsb.org/structure/6M3M) | 2020 |
| 2. | 6WKP | [Crystal structure of RNA-binding domain of nucleocapsid phosphoprotein from SARS CoV-2, monoclinic crystal form](https://www.rcsb.org/structure/6WKP) | 2020 |
| 3. | 6YI3 | [The N-terminal RNA-binding domain of the SARS-CoV-2 nucleocapsid phosphoprotein](https://www.rcsb.org/structure/6YI3) | 2020 |
| 4. | 6VYO | [Crystal structure of RNA binding domain of nucleocapsid phosphoprotein from SARS coronavirus 2](https://www.rcsb.org/structure/6VYO) | 2020 |
| 5. | 1SSK | [Structure of the N-terminal RNA-binding Domain of the SARS CoV Nucleocapsid Protein](https://www.rcsb.org/structure/1SSK) | 2004 |
| 6. | 2OFZ | [Ultrahigh Resolution Crystal Structure of RNA Binding Domain of SARS Nucleopcapsid (N Protein) at 1.1 Angstrom Resolution in Monoclinic Form.](https://www.rcsb.org/structure/2OFZ) | 2007 |
| 7. | 4UD1 | Structure of the N Terminal domain of the MERS CoV nucleocapsid | 2015 |
| 8. | 6KL2 | [Structure of the N-terminal domain of Middle East respiratory syndrome coronavirus nucleocapsid protein](https://www.rcsb.org/structure/6KL2) | 2020 |
| 9. | 3HD4 | [MHV Nucleocapsid Protein NTD](https://www.rcsb.org/structure/3HD4) | 2009 |
| 10. | 4LM9 | [Crystal structure of HCoV-OC43 N-NTD complexed with GMP](https://www.rcsb.org/structure/4LM9) | 2014 |
| 11. | 4J3K | [Structure of the N-terminal domian of human coronavirus OC43 nucleocapsid protein](https://www.rcsb.org/structure/4J3K) | 2013 |
| 12. | 2GEC | [Structure of the N-terminal domain of avian infectious bronchitis virus nucleocapsid protein in a novel dimeric arrangement](https://www.rcsb.org/structure/2GEC) | 2006 |
| 13. | 2BXX | [Crystal structure of the N-terminal domain of IBV coronavirus nucleocapsid. Native crystal form](https://www.rcsb.org/structure/2BXX) | 2005 |
| 14. | 2BTL | Crystal structure of the N-terminal domain of IBV coronavirus nucleocapsid | 2005 |
| 15. | 5N4K | [N-terminal domain of a human Coronavirus NL63 nucleocapsid protein](https://www.rcsb.org/structure/5N4K) | 2017 |
